# Opportunities and challenges in understanding complex functional materials

**DOI:** 10.1038/s41467-019-12422-z

**Published:** 2019-10-01

**Authors:** Andrew L. Goodwin

**Affiliations:** Department of Chemistry, University of Oxford, Inorganic Chemistry Laboratory, South Parks Road, Oxford, OX1 3QR UK

**Keywords:** Materials chemistry, Materials for devices, Condensed-matter physics

## Abstract

Understanding complex functional materials suffers from needing to capture structural features on many length scales. By quantitatively combining complementary experimental measurements, realistic models can now be generated. Here, I discuss the strengths and limits of this approach, but also advocate focusing on the interactions that drive structural complexity instead.

## Complexity in functional materials

The extent to which a material’s structure is simple or complex reflects the amount of information required to describe it. The structure of silicon, for example, is simple because it is captured entirely by a handful of structural parameters. Crystallographic symmetry is clearly crucial, because it projects the enormous number of degrees of freedom in the bulk onto extremely few microscopic descriptors. By contrast, relaxor ferroelectrics are complex because their compositions and structures vary over the nanometre length scale, and so any realistic model must contain the positions of many hundreds of thousands of atoms^1^. For relaxors, this complexity is considered responsible for their unusual dielectric properties. No model of a complex material is ever unique: different regions of the system will correspond to different individual sets of atomic coordinates. The best one can do is to make a model sufficiently large (and realistic) to capture all key features and correlations, and hence be representative of the whole.

Unsurprisingly, the atomic-scale structures of complex materials are difficult to determine. Crystallographic approaches are sensitive to anything periodic; i.e., the ‘average structure’. Local probes provide information about deviations away from this average, but different tools capture different many-body correlations, and so are sensitive to local structure in different ways. However expertly carried out, each individual measurement nonetheless provides an incomplete picture of the true structure. If understanding the function of complex materials truly depends on determining their atomic-scale structures, then the clear challenge is to develop and apply a self-consistent methodology for pulling together these various complementary measurements. This is the long-recognised objective of so-called complex modelling^2^. There is also the possibility, however, that the underlying physics of complex functional materials might be captured much more simply by focusing on interactions rather than structure.

## Hierarchical structure of a relaxor ferroelectric

Probably the most successful and thorough application of complex modelling to date is a recent study of the canonical (and ever controversial) relaxor ferroelectric PbMg${}_{1/3}$Nb${}_{2/3}$O${}_{3}$ (PMN)^3^. As for many complex materials, the average structure of PMN is described by a deceptively simple, high-symmetry unit cell. Yet its technologically relevant response to electric fields arises from deviations away from this high-symmetry structure, in the form of compositional variation and large-scale cation off-centering. The extent to which off-centre displacements are coupled to the non-random distribution of Nb${}^{5+}$ and Mg${}^{2+}$ ions, and the nature of structural inhomogeneities on the nanometre scale are both long-standing open questions.

**Fig. 1:**
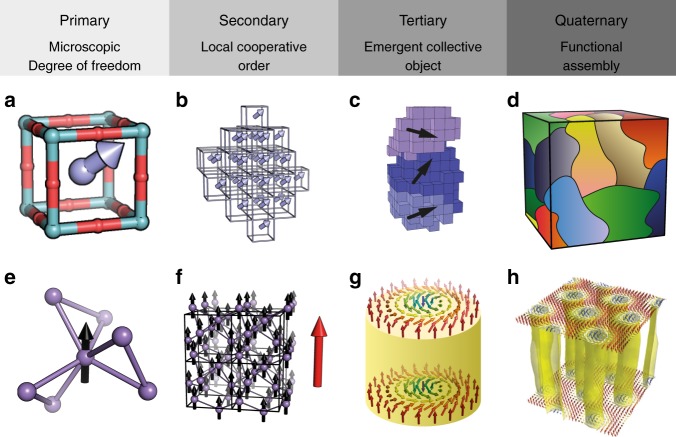
Hierarchical structures of complex materials. **a**–**d** The structure of PbMg${}_{1/3}$Nb${}_{2/3}$O${}_{3}$ relaxor ferroelectric determined in ref. ^3^. **a** At the unit-cell level, the cations are displaced from their high-symmetry positions (arrow) at the centre of the perovskite cages (balls and sticks), and these displacements are correlated with local composition. **b** Neighbouring unit cells tend to coalign their displacements, forming domains with a collective polarisation. **c** Collections of nearly aligned domains form larger structures consistent with the polar nanoregion (PNR) model discussed widely in relaxor ferroelectric literature^1^. **d** Finally, the bulk structure consists of a dense packing of these PNRs (here different PNR orientations are shown in different colours, with gradation reflecting the presence of nearly-aligned sub-domains). **e**–**h** The magnetic structure of a MnSi skyrmion crystal. **e** Magnetic moments of Mn atoms (black arrow) on the chiral MnSi lattice couple ferromagnetically to give (**f**) a net local magnetisation (large red arrow). **g** On larger length scales, the underlying lattice chirality causes this magnetisation to twist on itself, giving rise to a knotting of the magnetisation field known as a skyrmion. Here, arrows are coloured according to the orientation of the magnetisation field, and the yellow surface denotes a region of constant magnetisation surrounding the one-dimensional skyrmion core. **h** In the bulk, these skyrmions assemble into a crystal phase, which in turn can be observed and manipulated directly. Panel figure adapted from ref. ^13^. Reprinted with permission from AAAS

The real success of ref. ^3^ was to combine the complementary structural information contained within eight distinct experimental data sets—spanning neutron and X-ray scattering, diffraction and element-specific X-ray absorption measurements, real- and reciprocal-space normalisations, for both powder and single-crystal samples—to arrive at a single consistent structural model of PMN. For context, most previous complex modelling studies—whether of PMN or of entirely separate systems—have included at most two or three data sets. The step-change here was made possible by a recent optimised implementation of the complex modelling code rmcprofile^4^, which used the reverse Monte Carlo (RMC) algorithm to construct of a 320,000-atom representation of the PMN structure consistent with the entire ensemble of input data. The hierarchical nature of the PMN structure is illustrated and described in Fig. 1a–d.

## Making realistic models of complex materials

At face value, we now find ourselves with a powerful—if computationally and experimentally demanding—tool capable of determining the structures of materials of perhaps arbitrary complexity. If this approach proves robust, it could provide unparalleled insight into the microscopic nature of all sorts of complex systems: examples include battery materials, biominerals, photovoltaics, thermoelectrics and heterogeneous catalysts. As systems become trickier and trickier to characterise, we might simply add to our modelling algorithm more and more data sets of increasing quality and increasingly diverse sensitivity. Even for the case of PMN, one might eventually wish to include small-angle scattering, three-dimensional pair distribution function (3D-PDF) and/or spectroscopic measurements.

A key difficulty is always going to be that of knowing how much to trust the answer. I do not think it’s yet clear, for example, how large is the error bar on the hierarchical structure of PMN. Even if experimental uncertainties were propagated fully—and there are good reasons why it is difficult to do so for e.g., PDF measurements—determining the uncertainty in emergent features is an even greater and less well-defined challenge. Checking for consistency in independent instances of a complex modelling process is of course good practice. Yet consistency is a necessary-but-not-sufficient criterion for correct structure solution: stochastic methods such as RMC are biased towards configurationally accessible solutions, whether or not they are actually physical^5^. Deterministic alternatives, such as the Diffpy-CMI approach^6^, are also not immune from uniqueness problems, and suffer more generally from the need to identify ab initio a suitable nanostructure model. In particular, one never knows whether a more physical model may exist and account for the same experimental observables equally well (or indeed more accurately^7^).

An obvious means of correcting this bias so as to favour physical solutions is to exploit energy calculations during the modelling process. The conventional approach is to include simple empirical potentials e.g. to constrain bond lengths and angles^4^, but newer frameworks anticipate the possibility for incorporating calculations based on higher-level theory^8^. A particularly exciting opportunity is the interface with efficient machine-learned potentials that allow energy calculations with density functional theory accuracy even for large atomistic configurations^9^. Whatever methodology one might use, the relative likelihood $p$ of any two competing models (A and B, say) is in principle entirely determined by the difference in corresponding (free) energies $E$: ${p}_{{\rm{A}}}/{p}_{{\rm{B}}}=\exp [-({E}_{{\rm{A}}}-{E}_{{\rm{B}}})/{k}_{{\rm{B}}}T]$. Bayes’ theorem could then be used to weight experiment and theory (exactly) in the case that experimental uncertainties are properly handled. Given the recent developments in both experiment-driven and computation-driven complex structure solution, we now have the right mise en place for a truly integrated methodology that promises the most realistic structural models possible. Two particular challenges remain: one is the computational difficulty of accounting for the contribution of entropy (doable, but slow), and the second is the trickier problem that complex materials need not be equilibrium phases.

## Recovering simplicity: from structure to interaction

Yet all this effort is predicated on one fundamental assumption: that by knowing the structure of a complex material, we will better understand its underlying physics. No doubt this is true, at least in part, for PMN. Its domainlike architecture does indeed help explain the unusual response to external electric fields, even if the physics that drive this particular structure remain opaque. But what if there had been no domains? Interpreting atomistic models for complex materials is its own particular challenge: my personal experience is that it can be very difficult to find the right order parameter to reveal hidden patterns^10^, and one is never certain that any description is ever complete. Meanwhile, in the parallel field of liquids and amorphous materials, it is increasingly clear that collective measures (such as density or mean-squared displacement) are often a better descriptor of properties than are atomic coordinates^11^.

One alternative is to focus on interactions, rather than structure. This is generally the approach of direct modelling, whereby a given interaction model is used to drive (e.g.,) a Monte Carlo or molecular dynamics simulation, which in turn is assessed in terms of its ability to account for experimental observables^12^. The clear advantage is that the output is the physics itself: namely, how individual components of a system interact so as to produce the various effects observed experimentally.

There is a particular beauty in the finding that complex structures can emerge from simple interactions. Many examples are known, but Fig. 1e–h illustrates the specific case of skyrmion magnets. Here, a handful of ingredients—ferromagnetism, lattice chirality and antisymmetric exchange—give rise to a hierarchical ‘skyrmion crystal’ phase at specific combinations of temperatures and magnetic field^13^. So this ostensibly complex magnetic structure can be captured much more simply in terms of very few microscopic interactions. The system complexity is reduced fundamentally by shifting from atomic positions to atomic interactions. An obvious question is whether the hierarchical structure of PMN shown in Fig. 1a–d might also emerge from relatively simple interactions. Indeed an interaction-based model for PMN was developed in ref. ^14^, and the immediate community will no doubt wish to compare the results of that study with the recent RMC work. In particular, if it can be shown that both arrive at essentially equivalent real-space descriptions, then the structural complexity evident in Fig. 1a–d arises entirely from a combination of simple cation-ordering rules and the interactions between neighbouring Pb–O, Mg–O, Nb–O and O–O atom pairs. Having reduced the system to these few parameters, one might more straightforwardly assess how to vary these parameters to control the relaxor state.

In this context, perhaps the bigger challenge in complex materials science is to develop general methodologies for finding the right interaction model and corresponding theory that allow complex structures and their underlying physics to be understood as simply as possible. Whereas historical approaches have relied heavily on the personal insight of key individuals in the field^12^, machine-learning approaches might offer an opportunity to capture this insight and make it more widely available. One might also learn from the frustrated magnetism community, where mean-field theory is exploited to fit the data for complex magnetic states directly in terms of pairwise interactions, avoiding the generation of atomistic models altogether^15^. There seems an obvious opportunity to extend such methods to non-magnetic systems.

## Acknowledgements

The author gratefully acknowledges funding from the E.R.C. (Grant 788144).
